# Radiofrequency ablation and cryoablation of renal tumours

**DOI:** 10.3747/co.2007.113

**Published:** 2007-02

**Authors:** K.G. Kwan, E.D. Matsumoto

**Keywords:** Radiofrequency ablation, cryotherapy, renal cell carcinoma

## INTRODUCTION

With widespread utilization of noninvasive cross-sectional abdominal imaging, small solid renal masses are being found with increasing frequency 1. These small tumours are often discovered incidentally by abdominal ultrasound or computer tomography (ct). These incidentally discovered renal tumours are generally slower growing, are detected at an earlier stage, and are localized to the kidney 2,3. The triad of pain, hematuria, and palpable mass is now more the exception than the rule. Many patients now treated for renal cell carcinoma (rcc) are asymptomatic at presentation.

The radical nephrectomy has been the “gold standard” for the treatment of clinically localized rccs, but a shift has occurred toward treating small, incidentally found renal neoplasms in a nephron-sparing manner. Nephron-sparing techniques have been shown to offer oncologic and functional outcomes that are equivalent to those with radical nephrectomy for patients with renal tumours 4 cm or smaller in size 4–6.

Since the mid 1990s, the movement toward minimally invasive alternatives has meant the replacement of open surgery (radical or partial nephrectomy) with laparoscopic techniques and now with *in situ* ablative technologies 7,8. Ablative techniques offer advantages over extirpative techniques by reducing perioperative morbidity, shortening the hospital stay, promoting faster recovery, and importantly, potentially treating patients who are poor surgical candidates while preserving renal parenchyma 9,10.

Several ablative technologies have been investigated, among them, cryoablation (ca), radiofrequency ablation (rfa), microwave 11, high-intensity focused ultrasound 12,13, laser interstitial thermotherapy 14, microwave thermotherapy, and radiosurgery. The current outcomes with rfa and ca are promising, but long-term studies are ongoing to validate their oncologic efficacy and durability.

This overview briefly outlines advances in energy-ablative techniques for rcc and provides a synopsis of recent clinical studies of rfa and ca.

## RADIOFREQUENCY ABLATION

Radiofrequency ablation is a heat-mediated method of tissue destruction. The technology was initially developed for treating primary and metastatic liver lesions 15. Zlotta *et al.* first described the use of rfa as the primary treatment for small renal tumours in 1997 16. In recent years, rfa has become the most commonly used percutaneous ablative technique for rccs. Its use has been described in patients with small renal tumours who have poor renal reserve, multiple bilateral rcc in Von Hippel–Lindau, or hereditary rccs, or in those who are poor surgical candidates 17. Contraindications to rfa include an uncorrected coagulopathy, acute illness or infection, recent myocardial event, and poor life expectancy. Tumour factors predicting rfa failure include large tumours (larger than 4 cm) and tumours in the hilum or the collecting system.

Radiofrequency ablation works by transmitting a high-frequency electrical current through an electrode placed directly into the renal tumour. Alternating current delivered through the probe causes ions in the surrounding tissues to vibrate, creating frictional heat that results in heat-induced tissue damage. The mechanism of tissue destruction has been extensively reviewed 18.

At a molecular level, the heat generated by the high-frequency electrical current causes tissue destruction in three phases. Immediately post-ablation, molecular friction produces some combination of destruction of cellular structure, protein denaturation, membrane lipid melting, and cellular vaporization 18,19. Days after the ablation, coagulative necrosis with surrounding areas of cellular edema and inflammation is evident and leads to tumour destruction 19,20. The final evolution of the ablated tissue is re-absorption of the necrotic foci; the resulting fibrotic scar is non-enhancing on contrast imaging 21.

The success of tumour ablation with rfa depends on factors including probe temperature, generator power, temperature distribution, and targeting of the tumour 22–26.

For the cellular changes to occur as described earlier, temperatures above 50°C must be achieved. Earlier underpowered rfa generators have been replaced by new generators with upwards of 200 W that can consistently achieve temperatures above 100°C. However, temperatures higher than 105°C cause immediate vaporization and boiling of tissue, which creates gas bubbles, tissue carbonization, and eschar formation at the electrode. These effects increase impedance and reduce the extent of tissue ablation 20.

Many studies have aimed to achieve electrode temperatures between 50°C and 100°C. Innovations to reduce the impedance created at high temperatures include infusion of hypertonic saline into the target tissue during ablation. Electrodes are also designed in variously-sized configurations from single and multiple tines to expandable hooks. The radiofrequency may be applied using a temperature-based or impedance-based system - 24,27. Finally, rfa may be applied percutaneously or laparoscopically 7,21,28,29. Ultrasonography, ct, and magnetic resonance imaging (mri) have all been used to target lesions. Now, with the advent of fluoroscopic ct and open interventional mri, real-time ablation monitoring can be achieved.

Table i summarizes recently published studies on rfa. To date, Matsumoto *et al.* have reported the largest series: 109 tumours treated with percutaneous rfa^34^. The mean tumour size was 2.4 cm, and initial ablation was successful in 107 of the 109 tumours. A recurrence rate of 2.8% was reported during a mean follow-up of 19 months.

Similarly, Gervais *et al.* reported 100 tumours treated with percutaneous rfa 31. The tumour sizes ranged from 1.1 cm to 8.9 cm, with 9 tumours ranging in size from 4.0 cm to 8.9 cm and requiring multiple ablation sessions. All tumours smaller than 4.0 cm were ablated completely after a single course. These authors reported 79 lesions with no-contrast-enhancement ct at a mean follow-up period of 28 months.

The most recent study by Varkarakis *et al.* reports the ablation of 56 tumours with a mean tumour size of 2.2 cm. No residual tumour was detected on ct for 47 lesions at a mean follow-up time of 27 months 30.

The rfa procedure is not without complications. In a multi-institutional review of complications of cryoablation and radiofrequency ablation of small renal tumours, Johnson *et al.* reported 11 complications in 133 cases (8.2%) 40. The most commonly reported complication was pain and paresthesia at the site of electrode insertion for percutaneous rfa 40. Studies have also reported perinephric hematoma, obstruction at the ureteropelvic junction, ureter damage, ileus, and urine leak 41. Ureteropelvic junction scarring requiring nephrectomy has also been reported 42.

## CRYOABLATION

Cryoablation (or cryotherapy) involves freezing the target tissue with a cryoprobe *in situ.* The tumour is rapidly frozen, creating a cryolesion, which then undergoes necrosis over time and eventually heals by secondary intention. At a molecular level, the damage induced by the cryo-energy is two-fold 43. Initially, the freezing causes direct cell damage through rapid extracellular and intracellular freezing and ice formation. As a result, extracellular osmotic concentrations change, cell membranes become dysfunctional, and cell integrity is disrupted. Indirect cryotherapy-induced damage is caused by the impairment of tissue microvasculature by vasoconstriction, endothelial damage, microvascular thrombosis, and tissue ischemia 44,45. In addition, an immunologic response is also induced, resulting in further reaction to the neoplastic tissue 46. The success of cryoablation depends not only on the freezing and thawing cycles, but also on the lowest temperature that is reached and the duration for which that temperature is held.

Argon or nitrogen are the cryogens most commonly used for cooling to a temperature of –40°C, and their effect usually extends 1 cm beyond the lesion margin 47. Cell death in normal and neoplastic tissue occurs reliably at that temperature.

Cryoablation differs from rfa in that the extremes of temperature alone are not enough to completely destroy cells; the effects of delayed microvasculature failure are also required. The contraindications for cryotherapy are similar to those for rfa.

Cryoablation can be performed by open 48, laparoscopic, and percutaneous techniques 10,49,50. Unlike rfa, cryoablation requires real-time monitoring of the ice ball to ensure that the tumour is completely frozen and to minimize injury to the surrounding healthy tissue. To date, most cryoablation has been performed using laparoscopic techniques under ultrasound monitoring. An open or interventional mri has been used to permit real-time monitoring of the ice ball in a percutaneous approach 10. Recently, a group from Johns Hopkins published results of percutaneous cryoablation using real-time fluoroscopic ct 51.

Gill *et al.* published the first series of patients undergoing cryoablation in 1998 54. Table ii summarizes recent studies on cryoablation for small renal tumours.

Gill *et al.* 54,59 have reported the largest series of patients undergoing cryoablation to date. With 56 of 115 patients completing 3 years of follow-up at the time of publication, tumour size was reduced by 75%, and 2 patients showed malignancy in 6-month post-ablation ct-guided biopsy.

Cestari 57 *et al.* reported a series of 37 patients undergoing laparoscopic cryoablation. The mean follow-up time was 20.5 months, and 25 patients who underwent the postoperative ct-guided biopsies had negative results.

Most recently, Lawatsch *et al.* 52 reported a series of 59 patients undergoing laparoscopic cryoablation. Mean follow-up time was 26.8 months. Two recurrences were identified after cryoablation.

In a multi-institutional review of complications of cryoablation and rfa of small renal tumours, Johnson *et al.* reported complications in 139 cases (13.6%)^40^. As with rfa, pain and paresthesia at the site of probe insertion were the most commonly reported complications 40.

## CONCLUSION

With the number of incidentally detected small renal tumours increasing and minimally invasive techniques for treating those tumours becoming more common, investigators have turned toward energy-ablative technologies. In particular, small asymptomatic renal masses in older patients or in those who are poor candidates for surgery require treatment in a minimally invasive fashion with minimal morbidity.

Radiofrequency ablation and cryoablation both appear to be safe and effective methods of treating small renal tumours. Both can be deployed in a minimally invasive fashion, with percutaneous rfa being the least cumbersome approach. Percutaneous cryoablation requires real-time monitoring of the ice ball, and because of the need for open mri or fluoro-ct few centers have performed this technique to date. The early results appear promising; however, long-term follow-up data are needed to prove the efficacy and durability of both ablative technologies.

## Figures and Tables

**TABLE I:** Recent studies on radiofrequency ablation for renal tumours

				*Tumour type*				
*Reference*	*Patients (*n*)*	*Tumours (*n*)*	*Mean tumour size or range (cm)*	*Exophytic*	*Parenchymal, central, or mixed*	*Approach*	*Success onct(%)*	*Follow-up (months)*
Varkarakis *et al.,* 2005^30^	46	56	2.2	39	17	Perc	84 (47/56)	27
Gervais *et al.,* 2005^31^	85	100	1.1–8.9	67	33	Perc	99 (79/80)	28
Hwang *et al.,* 2004^32^	17	24	2.2	10	14	Lap=15	96 (23/24) Perc=9	13
Lewin *et al.,* 2004^33^	10	10	2.3	10	0	Perc	100 (10/10)	25
Matsumoto *et al.,* 2004^34^	91	109	2.4	N/A	N/A	Lap=46	98 (107/109) Perc=63	19
Ukimura *et al.,* 2004^35^	9	9	3.8	5	3	Perc	78 (7/9)	17
Zagoria *et al.,* 2004^25^	22	24	3.5	9	15	Perc	100 (<3 cm) 69 (>3 cm)	7
Farrell *et al.,* 2003^36^	20	35	1.7	22	13	Perc	100 (35/35)	9
Mayo–Smith *et al.,* 2003^37^	32	32	2.6	29	3	Perc	100 (32/32)	9
Roy–Choudhury *et al.,* 2003^38^	8	11	3.0	9	2	Perc	88 (7/8)	17
Su *et al.,* 2003^39^	29	35	2.2	28	7	Perc	100 (35/35)	9
Ogan *et al.,* 2002^29^	12	13	2.4	10	3	Perc	92 (12/13)	5
Pavlovich *et al.,* 2002^7^	21	24	2.4	13	11	Perc	79 (19/24)	2

ct = computed tomography; Perc = percutaneous; Lap = laparoscopic.

**TABLE II:** Recent studies on cryoablation for renal tumours

*Reference*	*Patients (*n*)*	*Mean tumour size or range (cm)*	*Approach*	*Follow-up (months)*	*Nephrectomy needed (*n*)*
Lawatsch *et al.,* 2006^52^	59	2.5	Lap	26.8	1
Bachmann *et al.,* 2005^53^	7	2.6	Lap	13.6	0
Gill *et al.,* 2005^54^	56	2.3	Lap	36	0
Silverman *et al.,* 2005^55^	23	2.6	Perc	14	0
Bassigiani *et al.,* 2004^56^	4	2.8	Perc	7	0
Cestari *et al.,* 2004^57^	37	2.6	Lap	20.5	0
Moon *et al.,* 2004^58^	16	2.6	Lap	9.6	0
Lee *et al.,* 2003^44^	20	2.6	Lap	14.2	0
Shingleton and Sewell, 2002^10^	20	3.0	Perc	9.1	0

Lap = laparoscopy; Perc = percutaneous.
